# Correction: Variations in anxiety and emotional support among first-year college students across different learning modes (distance and face-to-face) during COVID-19

**DOI:** 10.1371/journal.pone.0339630

**Published:** 2025-12-23

**Authors:** Flor Rocío Ramírez-Martínez, Maria Theresa Villanos, Sonam Sharma, Marie Leiner

The affiliation for the fourth author is not correct. Marie Leiner is not affiliated #1 but with #2: Department of Pediatrics, Texas Tech University Health Sciences Center, El Paso, Texas, United States of America.

## References

[1] Ramírez-Martínez FR, Villanos MT, Sharma S, Leiner M. Variations in anxiety and emotional support among first-year college students across different learning modes (distance and face-to-face) during COVID-19. PLoS One. 2024;19(3):e0285650. doi: 10.1371/journal.pone.0285650 38451887 PMC10919625

