# Supplementary material for: Variations in anxiety and emotional support among first-year college students across different learning modes (distance and face-to-face) during COVID-19
Source: PLoS One. 2024 Mar 7;19(3):e0285650. doi: 10.1371/journal.pone.0285650 (PMC10919625; doi:10.1371/journal.pone.0285650)
Supplement: Attachment — Submitted filename: PLOS comments v2.docx [file pone.0285650.s001.docx]

PONE-D-23-11873

Anxiety and emotional support among first-year college students during the transition from distance to face-to-face learning during COVID-19

PLOS ONE

Reviewer #1: The current study aimed to investigate anxiety and emotional support in a large sample of first-year college students transitioning from distance education to face-to-face learning during the COVID-19 pandemic.
The paper provides a good scientific contribution to the panorama of studies investigating the impact of COVID-19 pandemic on the general population's mental health, focusing on university students and their difficulties in building a productive and satisfying academic career, exacerbated during the pandemic. This study comprehensively analyzed the well-being trajectories in an extensive sample. The topic is interesting enough and qualitatively good to be published. However, some changes and explanations to improve the quality of the manuscript for his acceptance should be done.

Abstract section
In the 'abstract section' the Authors reported: "Data from 8659 freshman students were extracted from a university database". It would be useful to better contextualize the data collection, specifying the project's scope or initiative that promoted the data collection. Furthermore, the specification of the study courses to which the evaluated students belonged (humanities, mathematics, health, etc.) would be required.

Response:

We added more contextualization of the data collection in the abstract, specifying the time data was collected and adding participants' fields of study.

Introduction section
In the 'introduction section' the bibliography is appropriate and updated. It is known that university students reported high emotional distress, anxiety and depression, low self-esteem, concentration, and learning difficulties compared to the general population, all factors that often lead to an emotional circle with a significant impact on academic performance and social relationships (listed below). In addition, it is well known that medical students suffer an increased risk of depression compared to their peers currently enrolled in non-medical university courses, and the presence of depressive symptoms seems to occur as early as the 1st year of the student's medical education (listed below), especially in women.
The conditions of medical university students have also been a research focus in Italian surveys that found that medical students highlighted issues associated with anxiety and depression, emotional distress, low perceived quality of life, problems related to alcohol consumption, and the propensity to use substances as cognitive enhancers (listed below). In addition, the Authors do not describe, as one would expect, the impact on academia in terms of the social changes related to the COVID-19 outbreak. Distance education (DE) has replaced traditional face-to-face teaching, requiring great flexibility from university students. Additionally, home confinement compromised the possibility of fully experiencing university life, influencing academic study (i.e., delays in activities and digital platform use). The lockdown and restrictions related to the pandemic emergency probably increased students' difficulties, especially for first-year students who were starting a phase of important change for their life and future. For that reason, it would be helpful to mention some recent international studies on the impact of the COVID-19 pandemic on university students' mental health. Some authors focused on distance education's emotional and cognitive correlates during Covid-19 confinement, investigating samples of medical and health professions students on potential predictors of psychological distress and poor academic performance (listed below). Some authors focused on psychological, emotional, and cognitive correlates of social confinement in a sample of college students, integrating qualitative and quantitative analyses to identify potential predictors of traumatic distress during COVID-19. The Authors identified the thinking style "all or nothing" as the strongest predictor of traumatic distress in a sample of university students.
At the end of the introduction, the authors could report their study hypothesis.

Response:

We have reframed the introduction to include health science students and the social changes related to the COVID-19 outbreak in terms of the impact on academic context. We reviewed the suggested references focusing on the incoming college students from all disciplines and incorporated their findings.

Materials and methods section
It is not clear who promoted the survey. Is it a University psychological support Service? The Authors should make this clear.

Response:

In methods, under study design, we explained which program originated this data collection and interventions (not reported in this study). The program, called the Psychological Support Program, is briefly explained in the same section.

About the form information completed by each student, other characteristics such as, for example, informed consent, clinical variables (previous psychological problems and previous contacts with mental health services), and academic variables (off-course, off-site and current) were not taken into consideration of study. Authors should explain their choice or motivation.

We explained the motivation for collecting this information based on a statistical analysis of the services provided by the University Psychological Services program to students. This initiative to provide new students with programs to reduce their anxiety was designed to be anonymous and not linked to the student, given that return response is reduced when linked to personal information. At the same time the data collection took place, the services of the University Psychological Service were offered to students, including an app they can use to find resources and services if needed.

The tables are not exhaustive for accurate data presentation. The tables are disorganized, and the figures are unclear. The authors could explain the reason for this synthesis or choice.
The authors do not report socio-demographic data of the students' sample evaluated (i.e., the year of course attended, first-year or not, foreign or off-site students, previous psychopathological conditions...). About table 1, it contains insufficient data.

Response:

We have reformatted completely the results improving the tables and figures. The sociodemographic information that is available was added and reporting has been improved.

Discussion section
The limitations of the study and the conclusions section are not reported.

Response:

The limitations have been expanded.

The authors reported the need for university counselors as a useful strategy to help students cope with suffering and emotional difficulties to limit the potential structuring of psychopathological profiles over time. The authors could further discuss this aspect concerning the effectiveness that these university services have shown up to now.

Response:

We elaborated on the possibilities and challenges of university counseling services. We added the idea that preventive services, such as wellness programs, should be widespread and cannot be offered on a voluntary, non-credit basis because students do not participate as they should because they prioritize their efforts on classes that give them curricular credits.

In the bibliography section, not all references contain the 'doi'. Could the authors do a check?

Response:

DOI has been added if available.

Reviewer #2: This paper consider how anxiety and emotional support varied for entering first-year students across semesters with face-to-face and virtual instruction using repeated cross sections from Mexico. This is an important question, but I believe the manuscript needs significant revision for impact. My key concerns are as follows:

(1) The hypotheses were not clearly laid out in the introduction. I wasn't sure what the authors were testing until the methods section and even then it was not entirely clear.

Response:

We have addressed these issues in the introduction.

What are the theories this is based on?

Response:

We have added information regarding the theories addressed in the introduction

(2) While the introduction discusses the literature and motivates the general area of interest, I did not think it was clear about what gaps this current work would fill or how the current work would contribute to the body of knowledge

Response:

We have reframed the introduction to present the gaps the study covers and how the study contributes to the body of knowledge.

(3) More background on the context is needed to understand the findings. Do new students enter every semester and is spring semester entrance different from fall semester as it is in the US? If this survey is conducted during the first 2 weeks of an immersive program prior to experience the face-to-face or virtual instruction or hybrid that is being offered that semester, What happens during the immersive 2 weeks?

Response:

Context background has been added in the methodology where we elaborate on when, how, and why this study took place, including information about the city, the university and the support counseling program that was collecting the information.

Why would the type of instruction matter?

Response:

It seems based on the results of the study that matter for the students and the authorities. We feel that returning to classes increased the anxiety in the first semester of the return to face-to-face classes because it involved a change again. The study shows the levels and peak on anxiety that was observed and how the following semester the anxiety dropped. We feel is relevant in the sense that having that information show the need for interventions at the University Level. For the authorities this information motivated specific programs to help the students. Sometimes programs are implemented not knowing the specific problems of the students. Emotional support was a surprised for us. We expected similar levels across.

(4) Sample representativeness: what was the response rate and how did it compare to the population of entrants

Response:

We have included the total of participants that were virtually or face-to-face present at the time of requesting these surveys.

(5) How should we interpret findings across semesters. When anxiety and emotional support differs, is this due to different characteristics of entrants at different points in time or is it about the instruction?

Response:

The students are very similar in this public state university. We have expanded the demographic information; surveys are simultaneously collected when starting classes among a large sample of students. The study reports the association between anxiety and perceived social support among students with similar characteristics when the classes change from virtual to face to face. Emotional support decreases systematically from the first semester it was measured contrary to what we hypothesized about emotional support being maintained across the semesters.

Given that the survey is taken before the student has a chance to experience the university instruction, it seems strange to me to attribute trends as an effect of the type of instruction (there are so many reasons anxiety would be increasing and declining across cohorts during the pandemic).

Response:

There are some studies indicating that anxiety did change during the transition and that is what we hypothesize that will change based on the perception the students have of the virtual or face-to-face classes. It is true that it could be many reasons the large group felt similar anxiety in different times but is also possible that given that there are similar conditions between the students, the time of the data collection, etc. the transitions had an effect.

Maybe anxiety is just declining over time and less anxious students enter university at later periods of the pandemic?

Response: Anxiety is not declining in the different periods of the pandemic, but they peak when classes change from virtual to face to face and then there is a decrease. We added the negative correlation between anxiety and social support to clarify these points and feel that will be clearer.

I think the connection to the type of instruction is circumstantial at best. Maybe I am missing something about the institutional environment and/or maybe you are trying to make more of a claim about the high school instruction?

Response:
We have explained the institutional environment and the data collection to indicate our points we are proposing. There is an influence on how the students completed the previous level, whether virtual or face to face, and most likely the students completed several high school semesters virtually which might be the reason why they are more anxious to return to face-to-face classes. This study shows what happened right at the moment when they started college face-to face and we see the increase on anxiety peaks. Levels of social support declined systematically since measuring started, which was not expected.

(6) the description of the participants is unclear (longitudinal or repeated cross sections, entering first years, etc) until we get to the later procedures section. Can these combine to be clearer?

Response:

We have written new information that was not included and clarified the sections regarding the descriptions of the participants.
