# Supplementary material for: Variations in anxiety and emotional support among first-year college students across different learning modes (distance and face-to-face) during COVID-19
Source: PLoS One. 2024 Mar 7;19(3):e0285650. doi: 10.1371/journal.pone.0285650 (PMC10919625; doi:10.1371/journal.pone.0285650)
Supplement: Attachment — Submitted filename: Responses 3.docx [file pone.0285650.s002.docx]

Reviewer #2: Thanks for your response to my comments. In reading the new introduction, I see a better case made for the contribution and why the design might be useful for understanding the effect of virtual instruction. I also understand now the 2 hypotheses listed.

However my main remaining concerns are as follows:
(1) Who enters may vary across cohorts due to the anxiety already produced by the pandemic or other related factors

Response:

We agree that the “why” is unknown and that individual differences can occur in the cohorts. We are not aware of any individual factors that specifically contribute to an increase in anxiety levels for each of the students in these comparisons. To respond to this possible misleading assumption, we have included the possibility that other factors could contribute to anxiety and emotional support levels in an individual level in the limitations section. The mediating role of social support in anxiety among college students has been explored in various studies which include that perceived social support is not only directly associated with anxiety, depression, and insomnia among college students during the pandemic but also indirectly connected with these mental health variables via self-control. The fact that social support seems to decrease during the pandemic in this large sample might be an interesting finding.

In regard to the possible differences in the cohorts’ participants during this collection the university was very concerned about the levels of anxiety and implemented a wellness program to attempt to remediate these levels. There were no observable differences in the policies that affected students as far as it was reported, the admissions policies document has not been changed in 6 years until 2023. We reported this in the limitations.

(2) It is unclear why differences in anxiety and emotional support across cohorts of entering first-year students should be attributed to switch from virtual learning to face to face and not other factors

Response

As stated in the hypothesis, our objective was to present differences between the cohorts that occurred between semesters. In the case of anxiety, it increased during the semester they returned to face-to-face classes. To respond to this concern, we have included in the limitations the possibility that other factors could contribute to reducing or increasing the scores attenuated by the sample size.

The current framing seems misleading to me. We can see in the data that anxiety levels were higher among entering first year students when the university returned to face to face instruction than during semesters of virtual instruction. This could be due to the switch from virtual learning (though some students may have experience face to face or virtual learning previously). It may have nothing to do with the pandemic given that we do not have a pre-pandemic comparison, i.e., anxiety may always be higher in the face-to-face scenario.

Response:

We agree, although the framework presented does not consider the pandemic the cause of the anxiety levels or decrease in the emotional support, instead it is a global environmental factor that allowed us to observe possible differences during these semesters. Our point is that in similar conditions, considering the return to face-to-face classes, the student’s anxiety seemed to increase. We are presenting the differences observed in this population.

It could be something about other things that happened in Fall 2022, for instance, if the university changed who was admitted (it’s a much larger sample?) or other changes in policies that made students more anxious.

Response:

As far as we know, the university did not change anything globally and the number of students that applied and were accepted did not change in proportion in any of the semesters considered. The larger change we observed is registered statistically during the return to face-to-face classes. We have made changes in the document to report that no major changes in the admissions policies happened between these times. The fall of 2022 was a moment of return to partial normality and face-to-face classes that seem to have an influence on the anxiety levels perceived by the students, indeed we cannot report an effect based on this alone. However, the sample included is large and the overall anxiety levels seem to vary. The title of the study is now to show variations across semesters in pandemic times, without specifically pointing to returning to class as a cause, but instead to describe the large sample variations.

Emotional support is just declining over time and seems like it could be something not related to the switch from face-to-face to virtual, which just seems part of trend. I'd really like those distinctions to be clear in the description of the study/intro and in the discussion/limitations. The comparisons are interesting, but the different patterns related to anxiety and depression around the return to in-person could be driven by different factors among these.

Response:

Emotional support seems to follow a trend indeed and that is not related to the switch from face-to-face to virtual. We have added clarity in these distinctions in the sections included by the reviewer even changing the title to suggest that we are presenting variations, although we did not changed the hypotheses.

Additional context was useful, but I still did not see how we can think about comparison across the different cohorts from this. I still don’t understand whether the variation across Fall and Spring semesters is comparable in terms of selection into college—there do indeed appear to be differences according to Table 1.

Response:

The cohorts are composed of students who responded to these scales during immersion week. The differences in table 1 are due to variations in regard to the age, civil status, area of study and living conditions that are not unusual as the response was not mandatory. For example, in the studies by Andersen et al. (2022) and Wong et al. (2022) they examined the impact of college campus closures during the pandemic on students' sense of connectedness, engagement, and mobility, providing insight into the impact of pandemic-related disruptions on students' academic experiences and preferences. These studies showed population’s samples and provided valuable insights into the multifaceted effects of the COVID-19 pandemic on college students' study preferences. They highlight the importance of understanding and addressing the multiple challenges college students are facing during this unprecedented time.

For instance, did people put off going to college because it was not in person? Background on Covid situation during this period could also be useful for framing how to think about the timing. Part of the effect over time could be length of exposure to remote instruction prior to college. Was the country in lockdown, social distancing, what was the extent of remote education in high school?

Response:

We have added background information to highlight the situation in Mexico including lockdown and social distancing. Remote learning was extensive at the high school and college level from March 2020 to August 2022.

Reviewer #3: 1. The abstract can be further improved by specifying the key findings based on the scales used.

Response:

We have added key findings to the abstract in regard to the scales used.

2. Kindly include review of related research i.e Related Works in a sub section, before the methodology section.

We have added the subsection requested.

3. The presentation of the results need to simplify and easy to read/understand, as the level of statistical understanding might differs.

We have made reviews to the results to simplify them and make them easy to understand

Reviewer #4: This study investigates anxiety and emotional support in a very large sample of first-year college students.
The research methods and the H1, H2 etc are well defined as far as the aim of the study. There is a sufficient literature reference list and provides a good scientific evidence for the impact of COVID-19 pandemic on the general population's mental health, focusing on university students. The text is interesting, timely written and it is considered good to be published.
